# Vitamin D, Vitamin D Binding Protein, and Longitudinal Outcomes in COPD

**DOI:** 10.1371/journal.pone.0121622

**Published:** 2015-03-24

**Authors:** Louise J. P. Persson, Marianne Aanerud, Pieter S. Hiemstra, Annika E. Michelsen, Thor Ueland, Jon A. Hardie, Pål Aukrust, Per S. Bakke, Tomas M. L. Eagan

**Affiliations:** 1 Dept. of Thoracic Medicine, Haukeland University Hospital, Bergen, Norway; 2 Dept. of Medicine, Haukeland University Hospital, Bergen, Norway; 3 Dept. of Pulmonology, Leiden University Medical Center, Leiden, The Netherlands; 4 Research Institute of Internal Medicine, Oslo University Hospital Rikshospitalet, Oslo, Norway; 5 Section of Clinical Immunology and Infectious Diseases, Oslo University Hospital Rikshospitalet, Oslo, Norway; 6 K.G. Jebsen Inflammatory Research Center, University of Oslo, Oslo, Norway; 7 Institute of Clinical Medicine, University of Oslo, Oslo, Norway; 8 Dept. of Clinical Science, University of Bergen, Bergen, Norway; University of Dundee, UNITED KINGDOM

## Abstract

**Background:**

Associations between Vitamin D_3_ [25(OH)D], vitamin D binding protein (VDBP) and chronic obstructive pulmonary disease (COPD) are previously reported. We aimed to further investigate these associations on longitudinal outcomes.

**Methods:**

426 COPD patients from western Norway, GOLD stage II-IV, aged 40–76, were followed every six-month from 2006 through 2009 with spirometry, bioelectrical impedance measurements and registration of exacerbation frequency. Serum 25(OH)D and VDBP levels were determined at study-entry by high-performance liquid chromatography coupled with mass spectrometry and enzyme immunoassays respectively. Yearly change in lung function and body composition was assessed by generalized estimating equations (GEE), yearly exacerbation rate by negative binomial regression models, and 5 years all-cause mortality by Cox proportional-hazard regression.

**Results:**

1/3 of the patients had vitamin D deficiency (<20ng/mL) and a greater decline in both FEV1 and FVC, compared to patients with normal levels; for FEV1 this difference only reached statistical significance in the 28 patients with the lowest levels (<10ng/mL, p = 0.01). Neither 25(OH)D nor VDBP levels predicted exacerbation rate, change in fat free mass index or risk of death.

**Conclusion:**

Severe vitamin D deficiency may affect decline in lung function parameters in COPD. Neither 25(OH)D nor VDBP levels did otherwise predict markers of disease progression.

## Introduction

Chronic obstructive pulmonary disease (COPD) is one of the leading causes of morbidity and mortality worldwide [1]. The progression of COPD varies greatly between afflicted subjects, both in terms of lung function decline [2], exacerbation frequency [3], and development of comorbidities [4]. The reasons for this heterogeneity are largely unknown.

In cross-sectional studies, vitamin D deficiency (VDD) is common in COPD patients, and has been associated with lower lung function both in COPD patients [5–7] and in the general population [8]. Recently, a large longitudinal population based study found an association between lower plasma 25(OH)D levels with faster decline in lung function and a higher risk of COPD [9]. There are plausible mechanisms by which vitamin D could impact COPD pathogenesis, as the vitamin D receptor (VDR) is a nuclear transcription factor regulating the expression of more than 900 genes, many with immune functions [10]. Laboratory studies have found associations between vitamin D and a strengthened innate immune response against airway infections by the production of antimicrobial peptides (AMPs) together with enhanced macrophage phagocytic and chemotactic activity [11], improved lung development and lung tissue repair [12, 13]. On the other hand, vitamin D may down-regulate inflammation through decreased T-cell reactivity and pro-inflammatory response [14, 15]. Thus the impact of vitamin D on the pulmonary inflammatory response is yet unclear.

Vitamin D binding protein (VDBP) is the main carrier of vitamin D metabolites, and gene polymorphisms in the VDBP gene have been associated with COPD [5] and levels of 25(OH)D [5, 16]. It has recently been suggested that low levels of 25(OH)D do not indicate true vitamin D deficiency when levels of vitamin D binding protein also are low [16]. VDBP may also have functions relevant to COPD progression independent of vitamin D carriage such as macrophage activation [17] and recruitment of neutrophils [18].

Thus, there is a rationale for examining the association of circulating levels of 25(OH)D and VDBP on longitudinal COPD outcomes. To date, only the predictive value of circulating 25(OH)D has been reported in a longitudinal setting; three studies have examined decline in lung function, in which two were based on a general population [9, 19, 20], three studies assessed the risk for acute exacerbations (AECOPD) [21–23], two studies have examined mortality [22, 24], and none the decline in lean body mass, which is indicative of development of cachexia. In addition, the results from these studies have been conflicting, possibly due to lack of adjustment for supplementation in some studies [19–21, 23, 24] and possibly due to a selected study population, as some were post-hoc analyses from randomized trials on azithromycin in COPD [21, 24].

The Bergen COPD Cohort Study is a well-described 3-year cohort study of COPD patients and healthy controls from western Norway. The aim of the current study was to examine the association of circulating 25(OH)D and VDBP on 5 years all-cause mortality, number of AECOPD over three years, as well as change in lung function and lean body mass in COPD patients.

## Methods

### Study population

The Bergen COPD Cohort Study (BCCS) was conducted between 2006 and early 2010. The current study comprises 426 COPD patients enrolled in 2006, aged 40–76 years, and for whom vitamin D_3_ [25(OH)D] were measured at baseline. The patients had a clinical diagnosis of COPD, a smoking history of ≥10 pack-years, and a post-bronchodilation FEV_1_/FVC ratio <0.7 and FEV_1_ <80% predicted. The patients were recruited during stable state, and active autoimmune diseases or cancer within the last 5 years were cause for exclusion. The sampling of the study population in the BCCS and the data collection has been described in detail previously [6, 25].

All patients received written and oral information prior to inclusion, and provided signed consent. The regional committee for medical and health research ethics, Western Norway, approved the study (study number 165.08).

### Data sampling

A study physician examined all patients upon inclusion, and a structured interview regarding smoking habits, exacerbation history the last 12 months, respiratory symptoms, general medical history, and medication use was undertaken.

All patients performed spirometry after inhalation of 0.4 mg salbutamol, on a Viasys Masterscope (Viasys, Hoechberg, Germany).

Body composition was examined with bioelectrical impedance measurements in fasting state, with a Bodystat 1500 (Bodystat Ltd, Douglas, Isle of Man, UK). Fat free mass index (FFMI) was calculated as fat free mass in kilograms divided by the square of height in meters (kg/m^2^), and similarly fat mass index was calculated as fat mass (kg) divided by height squared (m^2^). Patients were classified as cachectic if FFMI <14 in women or <17 in men [26], or obese if FMI >13.5 in women or >9.3 in men [27]. Measurements were not performed in two patients due to non-consent, and not in a patient who had a pacemaker.

### Laboratory measurements

Peripheral venous blood was attempted sampled from all patients at baseline. Serum samples were coagulated at room temperature for 30–45 minutes, followed by centrifugation at 2500 x g for 15 minutes at 4°C. The serum was aliquoted and stored at −80°C in ultrafreezers.

A liquid chromatography double mass spectrometry (LC-MS/MS) [28] was used on serum never previously thawed to measure 25(OH)D, as described in detail previously [6]. The measurements of VDBP were performed on the same serum samples, during second thawing, by enzyme immunoassay (R&D systems, Stillwater, MN, USA). Intra- and inter- assay coefficients of variation were <10%.

### Missing values

For 2 patients we lacked serum for VDBP analyses, thus measurements of this parameter were available for 424 of the 426 patients. 29 patients only participated in the baseline visit. Among reasons for discontinuation were use of oral steroids, lung cancer revealed by early CT scans, withdrawal of consent and decease before any follow-up visits were performed. Thus, 426 patients were included in analyses on mortality, and 397 in analyses on later exacerbations, longitudinal change in lung function and body composition. For the other baseline variables there were no missing values.

### Outcome variables

For the subsequent three years follow-up, patients were invited to visits every 6 months. At all visits post-bronchodilation spirometry was performed. The study physician interviewed the patients at all visits, collecting a detailed history of COPD exacerbations since last visit with the aid of the patients' journal. The majority of patients were affiliated to our hospital; admission to our hospital was registered. Also, patients were instructed to contact the study-staff at periods at worsening of symptoms by a cell phone, and were offered a clinical examination by the study physician at the outpatient clinic, Dept. of Thoracic Medicine within 24 hours of contact, all of which would be recorded in the patients' journal. All exacerbations requiring treatment with either oral steroids or antibiotics were recorded during follow-up, and were included in the analyses. Bioelectrical impedance was measured yearly.

All citizens in Norway have a unique identification number given by birth, and all deaths are recorded in national registers that are linked to the electronic patient journals. After study completion, on the 25th of August 2011, the patients' records were screened for mortality. Date of death was recorded if applicable, whereas cause of death was not available for the current analyses.

### Statistical analyses

Whereas 25(OH)D had a normal distribution, the VDBP had an upper detection level of 500μg/mL, where higher levels were simply classified as higher (n = 2). Thus, non-parametric tests were applied to VDBP when examined as an outcome. A cut-off level of <20ng/mL, was used to define vitamin D deficiency (VDD). In addition sub-categories; less severe (10–20ng/mL) and severe deficiency (<10ng/mL) was used in analyses. VDBP was categorized in three categories and the ratio between 25(OH)D and VDBP was categorized in quartiles. Differences between patients with normal or deficient vitamin D levels, and low, medium or high levels of VDBP were examined with chi-square tests, t-tests or ANOVA.

For all-cause mortality, a Cox proportional-hazard model was fitted. A negative binomial regression model was used for analyses of annual exacerbation rate due to between-subject variation (overdispersion) [29] and the model was extended for panel data and allowed random effects to capture within subject associations over the time periods. The effects of 25(OH)D, VDBP and quartiles of their ratio on mean yearly change in FEV_1_, FVC and FFMI were investigated by generalized estimating equations (GEE) models [30] by including the outcome variables by time interaction and adjusting for baseline season, use of vitamin D supplements, sex, age, smoking, body composition, GOLD stage, and number of exacerbations (>2; yes or no) the last year before baseline. The mean number of FEV_1_ and FVC measurements included in the GEE models was 6 per subject, and for FFMI 3.7 per subject.

All analyses were performed with Stata version 13.1 (Statacorp LP, College Station, TX, USA).

## Results

COPD patients with VDD (<20ng/mL, n = 142) were more often current smokers, had more severe COPD, were more likely to be frequent exacerbators, and were more often either cachectic or obese compared to patients with non-deficient levels (n = 284) (Table 1). Female COPD patients were more likely to have higher levels of VDBP (p = 0.05), otherwise associations between VDBP and clinical characteristics were non-significant (Table 1).

**Table 1:** Baseline characteristics of the COPD patients in the study sample categorized by measured levels of serum vitamin D (25(OH)D) and Vitamin D binding protein (VDBP).

	25(OH)D > 20 ng/mL (n = 284)	25(OH)D < 20 ng/mL (n = 142)	p*	VDBP <200 μg/mL (n = 138)	VDBP 200–299 μg/mL (n = 240)	VDBP ≥ 300 μg/mL (n = 46)	p**
*Sex*, *%*			0.73				0.05
Women	40.5	38.7		37.2	37.7	59.1	
Men	59.5	61.3		62.8	62.3	40.9	
*Age*, *mean (SD)*	63.9 (6.8)	62.8 (7.0)	0.13	63.9 (7.4)	63.4 (6.8)	62.7 (5.9)	0.54
*Smoking*, *%*			<0.01				0.78
Ex	61.3	45.8		53.6	56.7	58.7	
Current	38.7	54.2		46.4	43.3	41.3	
*GOLD 2007 stage*, *%*			<0.01				0.80
II	56.0	27.5		44.2	46.7	54.4	
III	36.3	53.5		42.8	42.1	37.0	
IV	7.8	19.0		13.0	11.3	8.7	
*Number of exacerbations last year before inclusion*, *%*			0.02				0.12
0–1	85.6	76.8		83.3	84.2	71.7	
2+	14.4	23.2		16.7	15.8	28.3	
*Body composition*, *%*			<0.01				0.98
Normal	61.3	46.5		55.8	57.1	54.4	
Cachectic	26.8	31.7		29.7	27.1	30.4	
Obese	12.0	21.8		14.5	15.8	15.2	
*Taking calcium & vitamin D supplements*, *%*			0.27				0.11
No	95.8	97.9		96.4	97.5	91.3	
Yes	4.2	2.1		3.6	2.5	8.7	

* ttest for age, chi square for all other variables

** ANOVA for age, chi square for all other variabels.

**Fig 1:**
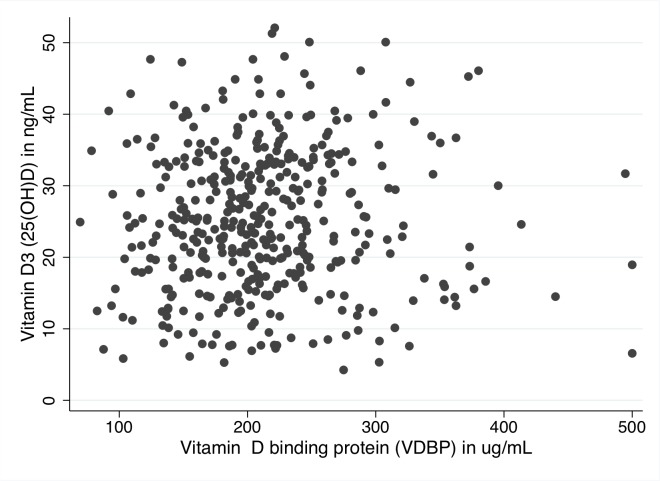
Scatterplot of measured 25(OH)D and Vitamin D binding protein in 426 and 424 COPD patients respectively.

A scatterplot of measured 25(OH)D and VDBP is presented in Fig. 1, and shows a low correlation between 25(OH)D and VDBP.

Thus, some patients with for instance low levels of 25(OH)D will have quite low levels of VDBP and therefore possibly a normal bioavailability of 25(OH)D, whereas other patients with low levels of 25(OH)D and high levels of VDBP could theoretically have a lower bioavailability of 25(OH)D.

During a follow-up time of 5 years, 69 patients out of 426 were deceased. 25(OH)D or VDBP levels at baseline in patients that died did not differ from patients still alive, generating non-significant hazard ratios and no predictive value of either 25(OH)D, VDBP or the ratio of 25(OH)D to VDBP on risk of death (Table 2).

**Table 2:** Predictive value of levels of 25(OH)D and Vitamin D binding protein on 5 years all-cause mortality (69 events) in 426* COPD patients (Hazard ratios with 95% confidence intervals).

	Adjusted only for season and supplements			Adjusted for season, supplements, sex, age, smoking, body composition, GOLD stage and number of exacerbations the last year before baseline		
	HR	95% CI	p	HR	95% CI	p
*Vitamin D*						
Per 10 ng/mL decrease	1.10	0.86, 1.41	0.46	0.95	0.71, 1.26	0.71
*Deficiency*						
>20 ng/mL	1			1		
10–20 ng/mL	1.62	0.94, 2.76	0.08	1.44	0.82, 2.55	0.21
<10 ng/mL	1.04	0.36, 2.94	0.95	0.80	0.26, 2.40	0.68
*Vitamin DBP*						
0–199 μg/mL	1			1		
200–299 μg/mL	0.88	0.53, 1.46	0.62	1.03	0.60, 1.75	0.93
300+ μg/mL	0.58	0.22, 1.54	0.27	0.76	0.28, 2.02	0.58
*Vitamin D3/ DBP ratio*						
Q4 (largest)	1			1		
Q3	1.01	0.51, 1.99	0.97	0.95	0.48, 1.90	0.89
Q2	1.22	0.64, 2.35	0.60	1.11	0.56, 2.19	0.76
Q1 (smallest)	0.86	0.40, 1.82	0.69	0.77	0.35, 1.68	0.51

* the analyses including vitamin DBP includes 424 COPD patients, of which 68 were later deceased.

**Fig 2:**
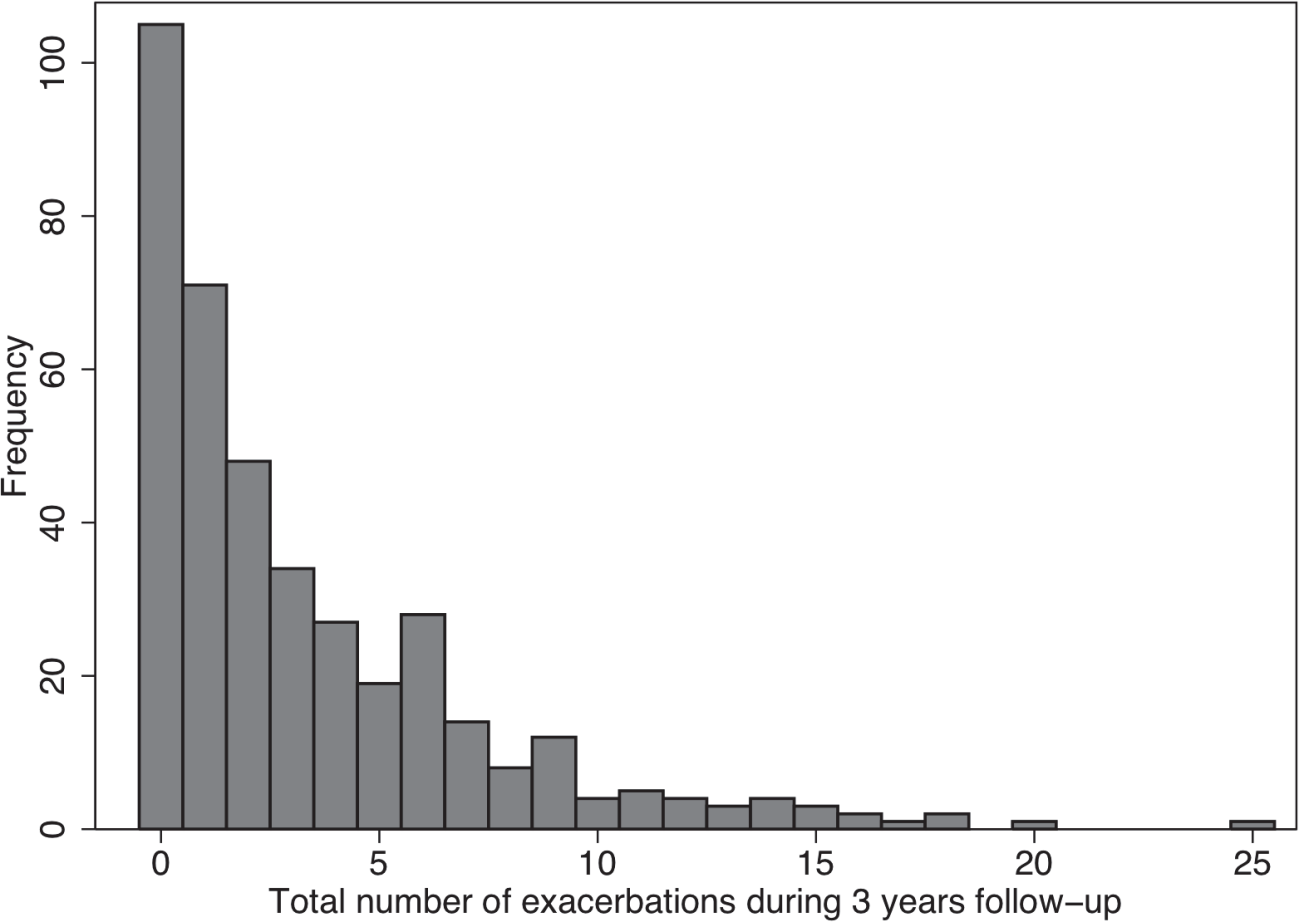
Total number of exacerbations during 3 years in 397 COPD patients.

There was a wide variety in exacerbation frequency, and its distribution is shown in Fig. 2.

Neither 25(OH)D nor VDBP levels or the ratio between 25(OH)D and VDBP were significant predictors of subsequent moderate or severe COPD exacerbations, where the incidence rate ratios approached 1 for most all categories regardless of adjustment (Table 3).

**Table 3:** Predictive value of levels of 25(OH)D and Vitamin D binding protein on subsequent yearly exacerbation rate in the 397 COPD patients (Incidence rate ratio with 95% confidence intervals) for whom we had follow-data on exacerbations.

	Adjusted only for season and supplements			Adjusted for season, supplements, sex, age, smoking, body composition, GOLD stage and number of exacerbations the last year before baseline		
	IRR	95% CI	p	IRR	95% CI	p
*Vitamin D*						
Per 10 ng/mL decrease	1.05	0.93, 1.19	0.46	0.96	0.85, 1.08	0.47
*Deficiency*						
>20 ng/mL	1			1		
10–20 ng/mL	1.09	0.82, 1.45	0.56	0.92	0.70, 1.19	0.52
<10 ng/mL	1.42	0.89, 2.27	0.15	1.09	0.72, 1.66	0.69
*Vitamin DBP*						
0–199 μg/mL	1			1		
200–299 μg/mL	0.96	0.74, 1.25	0.76	1.02	0.81, 1.29	0.88
300+ μg/mL	0.92	0.61, 1.40	0.71	0.96	0.66, 1.39	0.82
*Vitamin D3/ DBP ratio*						
Q4 (largest)	1			1		
Q3	1.03	0.74, 1.46	0.85	1.04	0.77, 1.39	0.82
Q2	1.12	0.79, 1.58	0.53	1.10	0.81, 1.49	0.55
Q1 (smallest)	0.98	0.68, 1.41	0.92	0.81	0.59, 1.12	0.20

Examining the effects of vitamin D deficiency on later change in lung function, it was found that COPD patients with vitamin D deficiency had a greater decline in both FEV_1_ and FVC per year, compared to patients with normal levels of 25(OH)D; both in the model only adjusted for season and supplements as well as in the fully adjusted model (Table 4). Vitamin DBP levels did not show any significant associations with change in either FEV_1_ or FVC (Table 4). COPD patients with smaller 25(OH)D/VDBP ratio had greater declines in FEV_1_, however only statistically significant for the second and third quartile, and not in the fully adjusted model.

**Table 4:** Predictive value of levels of 25(OH)D and Vitamin D binding protein on yearly change in lung function in 397 COPD patients estimated by generalized estimating equations (GEE).

	Adjusted only for season and supplements					Adjusted for season, supplements, sex, age, smoking, body composition, GOLD stage and number of exacerbations the last year before baseline			
	yearly change in FEV1 in % predicted	p	yearly change in FVC in % predicted		p	yearly change in FEV1 in % predicted	p	yearly change in FVC in % predicted	p
*Vitamin D*									
Per 10 ng/mL decrease	−0.107	0.28		−0.356	0.03	−1.05	0.26	−0.352	0.02
*Deficiency*									
>20 ng/mL	−0.894		−1.538			−0.898		−1.547	
10–20 ng/mL	−1.303	0.07	−2.757		0.001	−1.274	0.07	−2.747	0.001
<10 ng/mL	−1.869	0.02	−3.275		0.01	−1.885	0.01	−3.289	0.01
*Vitamin DBP*									
0–199 μg/mL	−0.853		−1.813			−0.853		−1.818	
200–299 μg/mL	−1.162	0.16	−2.204		0.52	−1.163	0.13	−2.205	0.49
300+ μg/mL	−1.058	0.55	−1.662		0.78	−1.049	0.54	−1.658	0.76
*Vitamin D3/ DBP ratio*									
Q4 (largest)	−0.706		−1.552			−0.709		−1.557	
Q3	−1.228	0.04	−1.621		0.87	−1.224	0.03	−1.618	0.89
Q2	−1.284	0.04	−2.365		0.07	−1.286	0.03	−2.381	0.06
Q1 (smallest)	−1.174	0.10	−2.301		0.08	−1.170	0.08	−2.300	0.08

**Fig 3:**
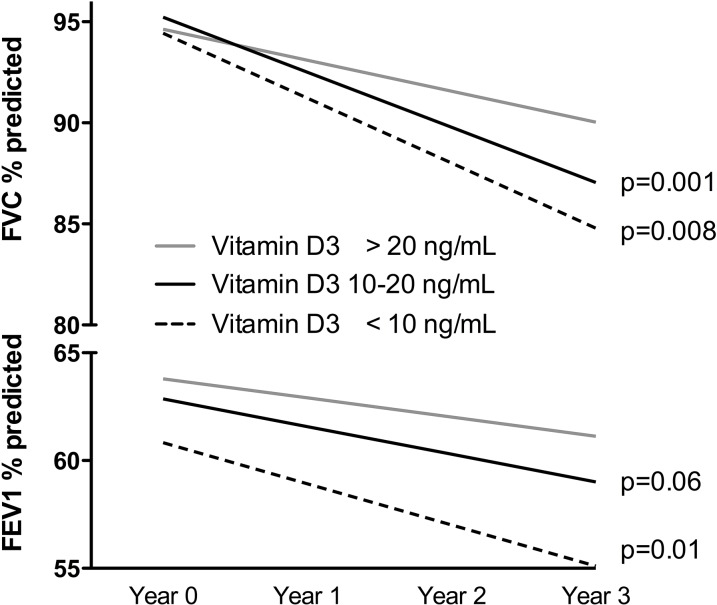
Yearly estimated change in FVC and FEV_1_ in percent predicted by levels of measured vitamin D at baseline.

To better show the meaning of the coefficients presented in Table 4, we graphed the estimated yearly change in lung function for patients with different levels of measured 25(OH)D at baseline, calculated from the coefficients from the multivariable regression analyses (Fig. 3).

Specifically, COPD patients with the lowest levels (<10ng/mL) of 25(OH)D had the largest decline in both FVC and FEV_1_ in percent predicted, whereas patients with intermediate deficiency (10–20ng/mL) also had significantly greater declines than patients without VDD.

As shown in Table 5, neither levels of 25(OH)D nor levels of VDBP impacted later change in FFMI, however there was a trend towards a smaller decline for the patients with the largest 25(OH)D/VDBP ratio (Table 5).

**Table 5:** Predictive value of levels of Vitamin D3 (25OH) and Vitamin D binding protein on yearly change in fat free mass index (FFMI) in 397 COPD patients estimated by generalized estimating equations (GEE).

	Adjusted only for season and supplements		Adjusted for season, supplements, sex, age, smoking, body composition, GOLD stage and number of exacerbations the last year before baseline	
	yearly change in fat free mass index (FFMI)	p	yearly change in fat free mass index (FFMI)	p
*Vitamin D*				
Per 10 ng/mL decrease	−0.026	0.22	−0.025	0.16
*Deficiency*				
>20 ng/mL	−0.043		−0.044	
10–20 ng/mL	−0.108	0.18	−0.109	0.12
<10 ng/mL	−0.025	0.84	−0.031	0.86
*Vitamin DBP*				
0–199 ug/mL	−0.086		−0.088	
200–299 ug/mL	−0.030	0.22	−0.031	0.14
300+ ug/mL	−0.054	0.65	−0.056	0.60
*Vitamin D3/ DBP ratio*				
Q4 (largest)	−0.012		−0.013	
Q3	−0.024	0.83	−0.026	0.78
Q2	−0.120	0.06	−0.119	0.03
Q1 (smallest)	−0.068	0.32	−0.069	0.25

## Discussion

In this longitudinal COPD cohort, in whom one third of the study sample had VDD, we were unable to find a predictive value of 25(OH)D on exacerbation rate, change in body composition or 5 years all-cause mortality. Meanwhile, we found a significant effect of deficient 25(OH)D levels on change in lung function parameters FEV_1_ and FVC, most convincingly for FVC. Alongside, VDBP was examined for the same outcomes but had no significant effect on neither of them.

Our study results must be interpreted within the limitations of having one single measurement of 25(OH)D, that only moderate to severe exacerbations were recorded, that only a small subgroup of patients (n = 28) had 25(OH)D levels <10ng/mL, and that we did not have data on gene polymorphisms in the VDBP gene. Also, the use of 25(OH)D as a substrate for vitamin D status may be less sensitive as it do not necessarily reflect local bioavailability/metabolism, as 1,25(OH)D would.

However, the strengths of this study includes adjustments for supplements, use of liquid chromatography double mass spectrometry for the measurement of 25(OH)D, the comprehensive follow-up of the study sample, and the hitherto unexamined potential influence of VDBP.

### 25(OH)D and mortality

Two previous population-based studies [31, 32] (with a median of 3.1 and 8.7 years of follow-up) have found higher mortality in subjects with very low 25(OH)D levels, a finding not replicated in our study on COPD patients.

We can not exclude that the lack of association between 25(OH)D and mortality in our study was due to lack of statistical strength. However, our results were in accordance with two recent studies; one 10-year follow-up study on 426 COPD patients originally included in a randomized trial with azithromycin [24] and another 2-year follow-up study on 356 COPD patients [22].

### 25(OH)D and exacerbation frequency

By modulating innate and adaptive immune responses, vitamin D may increase the production of antimicrobial peptides and autophagy of macrophages [11, 33] and thereby strengthen the host defense against infection. Clinical studies suggest an anti-infectious role of 25(OH)D in patients with bronchiectasis, and upper respiratory infection [34, 35].

To our knowledge, three previous studies with longitudinal design [21–23] and one randomized trial [36] have examined possible effects of 25(OH)D levels on infectious COPD exacerbations. These studies and our study report negative findings. One study was a secondary analysis of a randomized trial in 993 patients at risk of exacerbation [21], follow-up between the four studies varied between 1 to 3 years, and only one adjusted for supplements [22].

The mean number of exacerbations per patient-year in our study was identical to the average rate of 0.73 in the UPLIFT study [37] and slightly lower than the rates presented in the TORCH study (0.85) and the ECLIPSE study (0.85–1.34) [2, 38]. Thus, the combination of a high prevalence of vitamin D deficiency in our study population and a distribution of exacerbation frequencies representative for COPD patients in general, we believe, should generate statistical power enough to capture differences of clinical importance.

### 25(OH)D and lung function

A positive association between lung function and levels of 25(OH)D has been demonstrated in large population-based cross-sectional [8] and longitudinal studies [9]. This was also reproduced in cross-sectional studies on COPD that found an association between FEV_1_, FVC and 25(OH)D [7], or with FEV_1_ and 25(OH)D only [5, 6]. There have been few longitudinal studies to date on VDD and change in lung function. Kunisaki et al. followed 196 continuous smokers with COPD during 6 years and found no difference in baseline 25(OH)D levels between patients with rapid and slow decline in FEV_1_ [19]. This is in contrast to findings in a large population-based study where lower 25(OH)D levels was associated with faster decline in FEV_1_ in 1647 COPD patients [9]. Moreover, in a community-based sample of 626 elderly men followed for 20 years, the combination of current smoking and VDD was significantly associated with a faster decline in FEV_1_ [20]. Different from Afzal et al [9], we found a greater decline in both FVC and FEV_1_ per year in patients with severe VDD (<10ng/mL) compared to patients with normal levels. Similar observations were made for FVC (and with borderline significance for FEV_1_) in patients with less severe VDD (10–20ng/mL). Unsurprisingly, yearly change in the FEV_1_/FVC ratio was indifferent to 25(OH)D levels when examined in the same statistical model used in separate analyses on FEV_1_ and FVC (data not shown).

Vitamin D deficiency was more common in current smokers at baseline in our study sample. However, a post-hoc analysis, including an interaction term with 25(OH)D and smoking status, did not support previous findings [20], indicating effect modification of smoking on associations between 25(OH)D and lung function.

### 25(OH)D and body composition

The adverse event of a disproportional loss of fat-free mass, cachexia, is common, depicts higher risk for morbidity and mortality in COPD, and has been linked to increased systemic inflammation [4]. Vitamin D supplements have been shown to have a positive effect on lower-extremity function, muscle strength and balance in elderly [39] and higher levels of vitamin D have been associated with increased exercise capacity in COPD patients in a cross-sectional study [7]. Meanwhile, our data suggested no association between 25(OH)D and loss of fat free mass in this COPD population. Several other mechanisms are likely of greater importance than 25(OH)D considering the complexity of a cachectic condition. Our data does not exclude a possible association of 25(OH)D on muscle weakness or physical endurance in COPD; these issues should be addressed in future research.

### Vitamin D binding protein (VDBP)

Effects of VDBP were investigated on the same outcomes as for 25(OH)D, both as an independent factor and as a co-factor to vitamin D with the 25(OH)D/VDBP ratio. We calculated the 25(OH)D/VDBP ratio to theorize a substrate for bioavailable 25(OH)D, as total 25(OH)D levels may be impacted by the concentrations of VDBP [16]. However, taking VDBP into account when assessing VDD did not seem to add vital information to the status or effect of VDD in our study sample. Adding VDBP as a potential confounder in all multivariable regression models did not alter any effects of 25(OH)D, supporting this finding (data not shown).

We also hypothesized that functions of VDBP independent from 25(OH)D carriage may influence COPD progression, based on previous studies where VDBP was found to activate alveolar macrophages in sputum [17] and induce recruitment of neutrophils by increasing chemotactic activity of the complement peptide C5a [18]. Also, in cross-sectional data, higher systemic levels of VDBP have been associated with lower FEV_1_ in COPD patients [17], and higher sputum levels have been linked to increased airway inflammation and exacerbation frequency in patients with bronchiectasis followed for 3 years [34]. However these findings were not supported in our data. Likewise, VDBP showed no significant effect on mortality or change in body composition.

We were restricted by having serum VDBP levels only and are therefore unable to discuss the impact of local VDBP levels (sputum) on airway inflammation. The relationship between VDBP and COPD is previously described on the basis of gene polymorphisms in VDBP genes (GC) that show association with increased risk of COPD [5, 40] and low levels of 25(OH)D [5]. In our data, we cannot exclude a possible effect of different gene combination on outcomes examined.

In conclusion, we used the gold standard method to measure 25(OH)D levels and found that severe vitamin D deficiency predicted later decline in lung function in COPD patients, an important parameter of COPD disease progression.

25(OH)D or VDBP levels did otherwise not predict markers of disease progression. This may imply that the effects of vitamin D in COPD disease progression are so small as to be clinically less relevant, or that vitamin D deficiency is a later event rather than a potentially causal factor.

## Supporting Information

S1 Dataset Dataset used to build Table 1 and 2. (XLSX)

S2 Dataset Dataset used to build Table 3. (XLSX)

S3 Dataset Dataset used to build Table 4. (XLSX)

S4 Dataset Dataset used to build Table 5. (XLSX)
